# Survival analysis of three types of maxillary and mandibular bonded orthodontic retainers: a retrospective cohort

**DOI:** 10.1186/s12903-022-02202-5

**Published:** 2022-05-06

**Authors:** Navid Rezaei, Zahra Bagheri, Amin Golshah

**Affiliations:** 1 Department of Orthodontics, School of Dentistry, Kermanshah University of Medical Sciences, Shariati Street, Kermanshah, 67139546581 Iran; 2 Students Research Committee, School of Dentistry, Kermanshah University of Medical Sciences, Shariati Street, Kermanshah, 67139546581 Iran

**Keywords:** Survival, Orthodontic retainers, Maintenance

## Abstract

**Background:**

Retention is an important aspect of orthodontic treatment. This study aimed to analyze the survival of three types of maxillary and mandibular bonded orthodontic retainers.

**Methods:**

This retrospective cohort study evaluated the records of 118 orthodontic patients (90 females, 28 males, mean age of 22.34 ± 6.44 years) retrieved from a private orthodontic office. Data regarding the retainer failure, dental caries, unwanted tooth movements, maximum pocket depth (PD), and bleeding on probing (BOP) recorded at the follow-up sessions were extracted from patient records. Three types of retainer wires namely Bond-A-Braid, Orthoflex, and Retainium were compared regarding the abovementioned parameters. Data were analyzed by ANOVA, Chi-square, Monte-Carlo Chi-square, and Kruskal Wallis tests, the log rank test, and the Cox regression model.

**Results:**

The frequency of retainer failure was not significantly different between males and females, different age groups, or different treatment durations (P > 0.05). Wire fracture was the most common failure type in both the maxilla and mandible. Also, the frequency of failure was not significantly different between the maxillary and mandibular retainers (P > 0.05). The frequency of failure, and survival of the three types of retainer wires were not significantly different during a 5-year period (P > 0.05).

**Conclusions:**

The three types of orthodontic retainers had comparable survival rates. Their failure rate was not correlated with the age or gender of patients or the treatment duration.

**Supplementary Information:**

The online version contains supplementary material available at 10.1186/s12903-022-02202-5.

## Introduction

Retention is an important aspect of orthodontic treatment [1]. Patients undergoing orthodontic treatment expect a lifelong beautiful smile [2]. Thus, retention is imperative, and should be carefully monitored for several years or lifelong after orthodontic treatment [3]. Relapse refers to the tendency of the teeth to return to their baseline pretreatment position. In this process, the skeletal, dental, esthetic, and functional results can be reversed [4]. Thus, retention is a mandatory phase after orthodontic treatment to prevent relapse [5].

Adequate time should be allocated for gingival and periodontal remodeling, and reinstatement of function, as well as developmental changes to prevent relapse [4, 6]. Some other factors such as the muscle and soft tissue balance and parafunctional habits can also affect the orthodontic treatment outcome. Thus, risk of relapse exists for several years after treatment [7]. Accordingly, retainers are used to prevent relapse after orthodontic treatment [8, 9].

Fixed retainers were introduced for prevention of relapse of mandibular incisors in 1970 [10]. They are bonded to the lingual surface of the teeth, and are increasingly used by orthodontists since they do not compromise esthetics and are easy to use by patients for a long period of time [11, 12].

The rate of postoperative relapse is variable and unpredictable. The optimal efficacy of bonded lingual retainers for stabilization of the new position of lower incisors has been confirmed in the long-term [5]. However, complications such as retainer failure may occur [1]. Failure of bonded retainers may vary from simple separation of wire from one tooth to debonding of the entire length of the wire from the teeth, leading to retainer loosening [13]. Three types of failures may occur in bonded retainers: (I) fracture of the wire, (II) debonding at the wire-composite interface, (III) and debonding of adhesive at the enamel-composite interface [12, 14, 15]. Failure at the adhesive-wire interface is less common, while debonding at the tooth-adhesive interface commonly occurs, and is the most frequently reported mode of failure [16]. The rate of failure at the tooth-adhesive interface is reportedly 3.5–53% for metal retainers and 11–51% for fiber retainers [17–20]. Excessive masticatory forces due to eating hard foods is the most common cause of debonding at the tooth-adhesive interface [13].

According to a systematic review by Iliadi et al. [13] on the failure rate of different retainers, sufficient information is not available to reach a definite conclusion regarding the best type of retainer in terms of low failure rate; thus, selection of an appropriate retainer remains a subjective matter. According to Iliadi et al. [13] conclusive evidence is not available regarding the superiority of a particular type of retainer wire.

Irrespective of the location and severity of failure, a failed retainer should be repaired, because failure can lead to plaque accumulation, tooth discoloration, caries development, or unwanted tooth movements [3]. The teeth separated from the retainer may move and lead to unexpected consequences such as torque change between two incisors, movement of canine tooth in opposite direction, or gap formation between the incisors, causing esthetic problems for the patients and necessitating retreatment. Thus, periodic examination of retainers is imperative during the retention period [21].

Different orthodontic wires, adhesives, and bonding techniques have been compared for fixed retainers. A wide range of failure rates has been reported for each type of bonded retainer [13]. The reported failure rate for stainless steel retainer wires bonded only to canine teeth ranges from 13 to 37.7% [14, 22–24]. On the other hand, the failure rate of retainers bonded to the six lower incisors ranges from 9 to 14% [15, 25]. The failure rate of multi-stranded retainer wires (which have recently gained increasing popularity) ranges from 8.8 to 46% [15, 17, 19, 22, 25]. For resin fiber retainers, the reported failure rate ranges from 11 to 71%, and risk of failure of fixed maxillary retainers, irrespective of their wire type, is higher than that of mandibular retainers [17, 19, 26].

Despite the availability of several retention protocols [2, 27, 28], there is shortage of high-quality evidence regarding the best type of fixed retention [8, 13, 29], and no consensus has reached regarding the superior efficacy of a particular type of retainer wire over the other types [4]. Thus, this study aimed to compare three types of bonded retainers in terms of their survival rate.

## Methods

This retrospective cohort study evaluated patient records retrieved from a private orthodontic office. The sample size was calculated to be 29 in each group according to a previous study by Kocher et al. [21] assuming the hazard ratio of fracture of 0.027″ TMA wire to 0.022 × 0.016″ braided SS wire to be 0.42, patient ratio in the group to be P = 0.534, outcome probability of d = 0.5, type I error (alpha) of 0.05, type II error (beta) of 0.1, study power of 90%, and accuracy of 3.2 in fracture occurrence, using R software (R Core Team, Vienna, Austria) and Trial Size package.

### Participants

Records of orthodontic patients who had completed their orthodontic treatment and required retainers according to the professional opinion of their orthodontist were included in this study. The demographic information of patients such as their age and gender was recorded in a checklist. Written informed consent was obtained from each patient. The STROBE guidelines [21] for reporting of observational studies were followed. The study was approved by the ethics committee of Kermanshah University of Medical Sciences (IR.KUMS.REC.1399.1039).

The inclusion criteria were fixed orthodontic treatment of both the maxilla and mandible by the same orthodontist, having 2–4 mm of overbite after completion of treatment, and placement of maxillary and mandibular retainers immediately after the completion of active orthodontic treatment. No age restriction was applied [21]. The exclusion criteria were applied at two phases of (I) patient enrollment and (II) follow-up. The exclusion criterion applied at the patient enrollment phase was the syndromes affecting the dentomaxillofacial region. The exclusion criteria applied at the follow-up phase included orthodontic retreatment, retention with a retainer wire other than the three types evaluated in this study, different types of retainers used for the maxilla and mandible, changing or repairing the retainer during the follow-up period, and removal of the maxillary or mandibular retainer for prosthetic restorations.

**Fig. 1:**
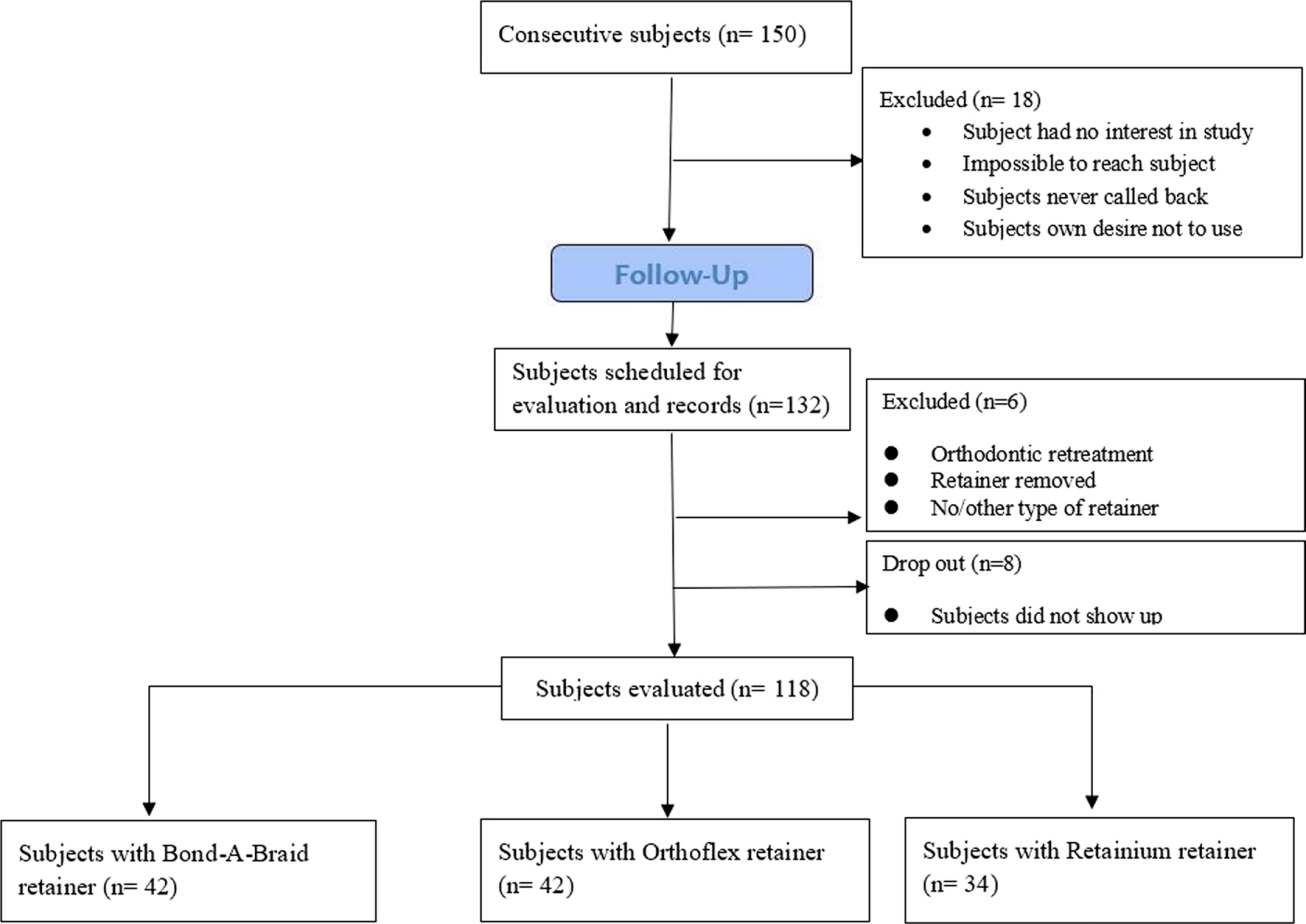
Flow diagram of the study

All patients were recalled at 2 weeks and 1, 3 and 6 months after retainer placement and annually thereafter. At each follow-up session, data regarding the retainer failure, dental caries, and gingival health were recorded in patient records. A final follow-up session was scheduled for patients in whom over 5 years had passed since their bracket debonding. Figure 1 shows the flow diagram of the study.

### Retention protocol

Three types of retainer wires were evaluated and compared in this study:

1. 0.026 × 0.010-inch Bond-A-Braid® (Reliance Orthodontic Products, Itasca, IL, USA)
2. 0.038 × 0.016-inch Ortho-Flex-Tech® (Reliance Orthodontic Products, Itasca, IL, USA)
3. Reliance Retainium® Superior Brand Lingual Retainer Wire (Reliance Orthodontic Products, Itasca, IL, USA)

The type of retainer wire for each patient was selected upon completion of orthodontic treatment by using a table of random numbers. The retainers were bonded to the six anterior teeth (to eight teeth in case of extraction) by an orthodontist in a standard manner. The surface of the teeth was cleaned by a low-speed hand-piece, rubber cup, and non-fluoridated pumice paste. The enamel was then etched with 37% phosphoric acid for 30 s, and after rinsing and drying, Transbond XT bonding agent (3 M Unitek, Monrovia, CA, USA) was applied on the enamel of each tooth and cured for 5 s. The retainers were positioned on the tooth surface using high-viscosity composite paste (3MTMFiltekTMsupreme Flowable restorative) and cured.

### Data collection

Clinical examination in the final follow-up session included assessment of the status of the retainers, and gingival and periodontal health [by assessment of maximum pocket depth (PD), bleeding on probing (BOP), and unwanted tooth movements] [30]. PD was measured at the lingual surface of each tooth, and maximum depth was recorded. A UNC-PCP15 color-coded probe (Hu-Friedy, Rotterdam, Netherland) was used for this purpose. After measuring the PD, the lingual sites were inspected for the presence/absence of BOP. Due to the low number of bleeding sites in patients, we reported positive (+) BOP for each arch if at least one site in the respective arch showed BOP, and vice versa for negative (–) BOP. Photographs were also taken, and impressions were made by an orthodontist. After the final follow-up of patients, the aforementioned second-phase exclusion criteria were applied.

Retainer failure was the primary outcome measure in this study. The time and type of retainer failure were also recorded. On each follow-up session, the status of the retainer was assessed by an experienced orthodontist and scored as follows:

- 0: Intact bonding
- 1: Debonding of the entire retainer
- 2: Fracture of the wire
- 3: Debonding of the wire
- 4: Composite damage
- 5: The retainer had been replaced with a new retainer
- 6: No retainer present
- 7: Several fractures at several locations

All assessments were performed by a calibrated orthodontist. The data were collected in a datasheet and statistically analyzed.

### Statistical analysis

The Kolmogorov–Smirnov test was applied to analyze the normality of data distribution. Since the data were normally distributed, ANOVA was used to compare the age and duration of treatment among the study groups. The Chi-square and Monte-Carlo Chi-square tests were applied to analyze the demographic variables and BOP. The Kruskal–Wallis test was used to compare the maximum PD of the study groups. The log rank test and the Cox regression model were also applied to assess the survival rate of the retainers. Data were statistically analyzed using STATA version 14 at 0.05 level of significance.

## Results

A total of 118 patients including 90 (76.3%) females and 28 males (23.7%) were evaluated in this study with a mean age of 22.34 ± 6.44 years. The Chi-square test showed no significant difference in survival of retainers in males and females neither in the maxilla (P = 0.582) nor in the mandible (P = 0.754). ANOVA showed no significant difference in survival of retainers in different age groups neither in the maxilla (P = 0.090) nor in the mandible (P = 0.080). No significant difference was noted among the groups in duration of treatment neither in the maxilla (P = 0.154) nor in the mandible (P = 0.300).

Table 1 presents the type of first failure in the maxilla.

**Table 1:** Type of first failure in the maxilla

Jaw	Type of first failure	Retainium			Orthoflex			Bond-A Braid			Total
		Female	Male	Total	Female	Male	Total	Female	Male	Total	
Maxilla	0: Intact bonding	10	2	12	10	1	11	8	2	10	33
	1: full retainer out and rebonded	0	0	0	0	0	0	0	0	0	0
	2: Fracture of the wire	17	5	22	11	10	21	22	6	28	71
	3: Detachment at the wire-composite interface or adhesive-enamel	0	0	0	0	0	0	0	0	0	0
	4: Composite damage	0	0	0	2	1	3	1	0	1	4
	5: retainer replaced by new retainer	0	0	0	1	0	1	0	0	0	1
	6: No retainer in situ at T3	0	0	0	0	0	0	0	0	0	0
	7: Multiple failures at the same time	0	0	0	4	0	4	1	1	2	6
Mandible	0: Intact bonding	15	3	18	13	7	20	21	6	27	65
	1: Full retainer out and rebonded	0	0	0	0	0	0	0	0	0	0
	2: Fracture of the wire	8	3	11	14	2	16	10	1	11	38
	3: Detachment at the wire-composite interface or adhesive-enamel	0	1	1	0	0	0	0	0	0	1
	4: Composite damage	2	0	2	1	1	2	0	0	0	4
	5: Retainer replaced by new retainer	0	0	0	0	0	0	0	0	0	0
	6: No retainer in situ at T3	0	0	0	0	0	0	0	0	0	0
	7: Multiple failures at the same time	0	0	0	2	1	3	1	1	2	5

Assessment of the failures of the three wire types indicated that in use of Retainium wire, the maximum and minimum frequency of failures in the maxilla occurred in the right canine and right central incisor, respectively. In the mandible, the maximum and minimum frequency of failures occurred in the right central incisor and left lateral incisor and canine teeth, respectively. In Orthoflex wire, the maximum and minimum frequency of failures in the maxilla occurred in the left canine and left second premolar, respectively. In the mandible, the maximum and minimum frequency of failures were recorded in the right lateral incisor and canine, and left canine and second premolar, respectively. In Bond-A-Braid wire, the maximum frequency of failures in the maxilla occurred in the left lateral incisor while the minimum frequency was recorded in the right central incisor. In the mandible, the maximum and minimum frequency of failures were recorded in the left central incisor, and canine and first and second premolars, respectively.

Table 2 compares the frequency of failures among the study groups in the maxilla and mandible. As shown, no significant difference was noted in the frequency of failures among the study groups neither in the maxilla (P = 0.280) nor in the mandible (P = 0.285).

**Table 2:** Frequency of failures in the maxilla and mandible in the study groups

				Number of failures				P value^†^
				Mean	Median	Percentile 25	Percentile 75	
Jaw	Maxilla	Retainer	Retainium	1.06	1.00	0.00	2.00	0.280
			Orthoflex	1.05	1.00	0.00	2.00	
			Bond-A-Braid	1.32	1.00	1.00	2.00	
	Mandible	Retainer	Retainium	0.53	0.00	0.00	1.00	0.285
			Orthoflex	0.76	1.00	0.00	1.00	
			Bond-A-Braid	0.53	0.00	0.00	1.00	

^†^ Kruskal–Wallis

**Fig. 2:**
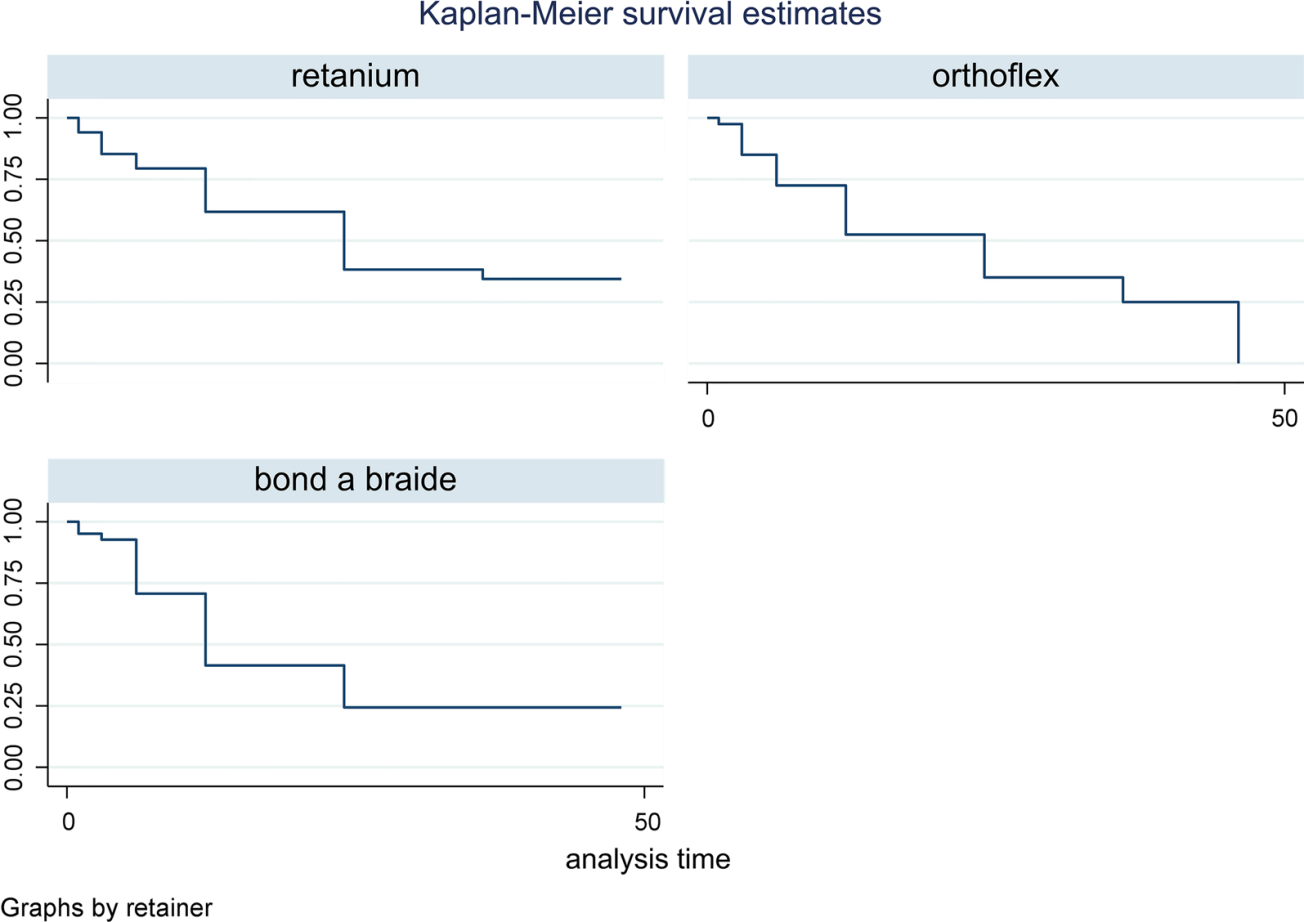
Kaplan–Meier estimator plot for the survival of different maxillary retainers

With respect to the survival rate of different retainer wires in the maxilla, Table 3 indicated no significant difference in the survival rate of different maxillary retainer wires (P = 0.432). Figure 2 indicates the Kaplan–Meier estimator plot for the survival of different maxillary retainers.

**Table 3:** Descriptive statistics regarding the survival rate of different retainer wires in the maxilla

Retainer	Time at risk	Event observed	Incidence rate	No. of subjects	Survival time			P value^†^
					25%	50%	75%	
Retainium	755	22	.029	34	12	24		0.432
Orthoflex	740	29	.039	40	6	24	46	
Bond-A-Braid	719	31	.043	41	6	12	24	
Total	2214	82	.037	115	6	24	46	

^†^ Log-rank

**Fig. 3:**
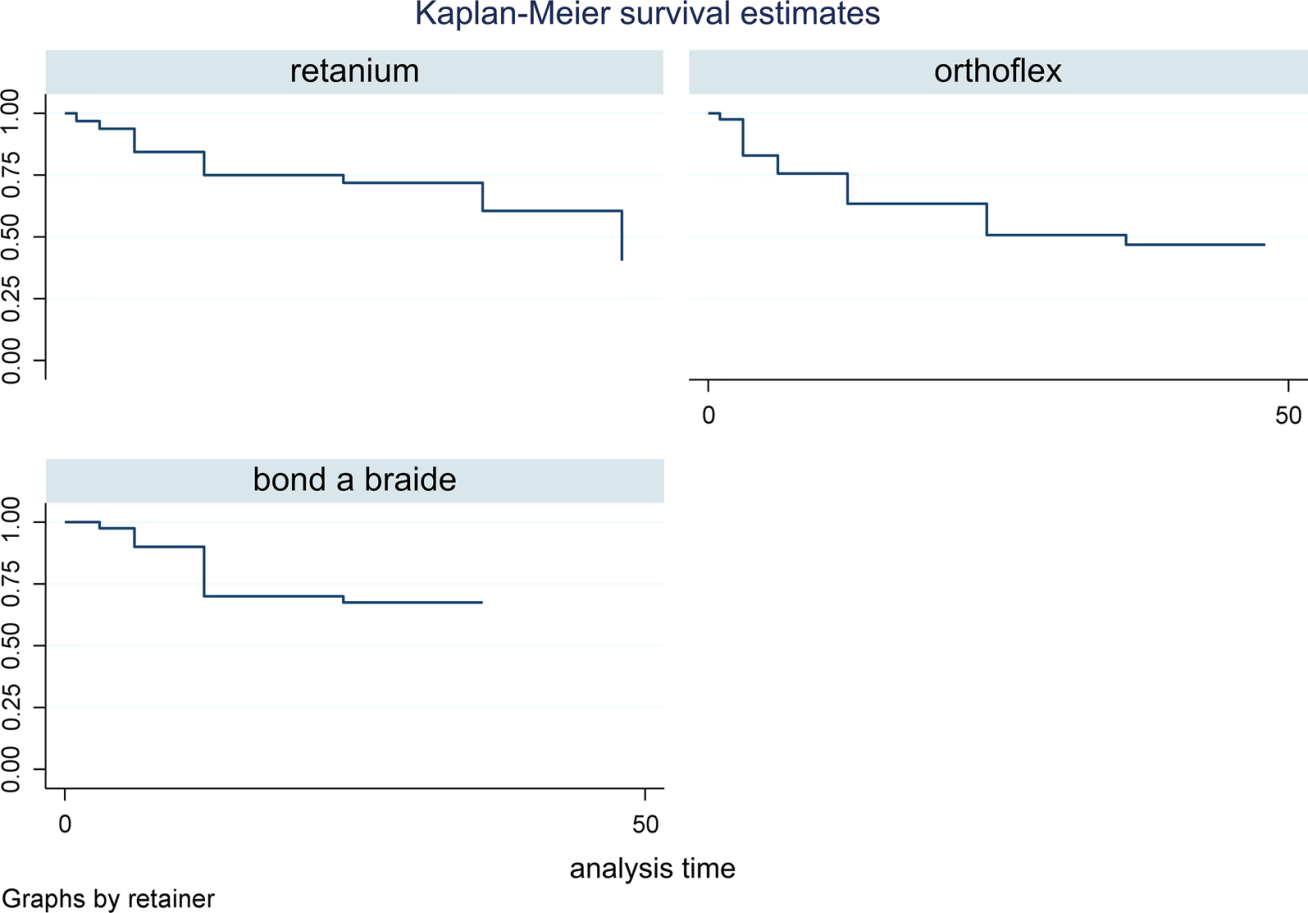
Kaplan–Meier estimator plot for the survival of different mandibular retainers

As shown in Table 4, no significant difference was noted in the survival rate of the retainer wires in the mandible (P = 0.195). Figure 3 indicates the Kaplan–Meier estimator plot for the survival of different mandibular retainers.

**Table 4:** Descriptive statistics regarding the survival rate of different retainer wires in the mandible

Retainer	Time at risk	Event observed	Incidence rate	No. of subjects	Survival time			P value^†^
					25%	50%	75%	
Retainium	934	14	.015	32	12	48		0.195
Orthoflex	889	21	.024	41	12	36		
Bond-A-Braid	1017	13	.013	40	12			
Total	2840	48	.017	113	12	48		

^†^ Log-rank

The Cox regression model for the assessment of the effect of type of retainer wire on its survival in the maxilla and mandible revealed no significant difference in the survival rate of different retainer wires neither in the maxilla nor in the mandible (Tables 5 and 6, P > 0.05).

**Table 5:** Cox regression model for assessment of the effect of type of retainer wire on its survival in the maxilla

		Haz. ratio	95% conf. interval		P value
			Lower	Upper	
Retainer	Retainium (ref)	1	–	–	–
	Orthoflex	1.223	.687	2.177	0.493
	Bond-A-Braid	1.351	.774	2.356	0.290
Age	1.000	.966	1.036	0.964	
Sex	Female (ref)	1	–	–	–
	Male	1.245	.764	2.027	0.378

**Table 6:** Cox regression model for assessment of the effect of type of retainer wire on its survival in the mandible

		Haz. ratio	95% conf. interval		P value
			Lower	Upper	
Retainer	Retainium (ref)	1	–	–	–
	Orthoflex	1.450	.721	2.913	0.296
	Bond-A-Braid	.815	.378	1.757	0.602
Age	.994	.949	1.040	0.800	
Sex	Female (ref)	1	–	–	–
	Male	.826	.409	1.667	0.594

With regard to BOP (Table 7), no significant difference was noted in BOP among the study groups neither in the maxilla (P = 0.671) nor in the mandible (P = 0.856).

**Table 7:** Comparison of BOP among the study groups in the maxilla and mandible

		Retainium		Orthoflex		Bond-A-Braid		P value^†^
Jaw	BOP	Count	Column N %	Count	Column N %	Count	Column N %	
Maxilla	BOP+	2	5.8	4	9.5	2	4.7	0.738
	BOP−	32	94.2	38	90.5	40	95.3	
Mandible	BOP+	3	8.8	3	7.1	2	4.7	0.897
	BOP−	31	91.2	39	92.9	40	95.3	

^†^Monte Carlo Chi-square test

With regard to PD (Table 8), no significant difference was noted in PD among the study groups neither in the maxilla (P = 0.646) nor in the mandible (P = 0.623). The difference in plaque index was not significant in the maxilla (P = 0.671) or the mandible (P = 0.856) either.

**Table 8:** Comparison of PD among the study groups in the maxilla and mandible

				Maximum pocket depth				P value^†^
				Mean	Median	Minimum	Maximum	
Jaw	Maxilla	Retainer	Retainium	2.00	2.00	1.00	4.00	0.646
			Orthoflex	2.29	3.00	1.00	4.00	
			Bond A Braid	2.18	2.00	1.00	3.00	
	Mandible	Retainer	Retainium	2.00	2.00	1.00	4.00	0.623
			Orthoflex	2.21	3.00	1.00	4.00	
			Bond A Braid	2.18	2.00	1.00	3.00	

^†^Kruskal–Wallis

## Discussion

This retrospective cohort study analyzed the survival rate of three types of fixed orthodontic retainer wires. The results indicated no significant difference in the survival rate of different retainer wires in the maxilla or mandible. The three groups were standardized in terms of gender and mean age of patients. Also, duration of treatment was not significantly different among the three groups, which increases the reliability of the results. The failure rate of orthodontic retainers had no significant correlation with the type of wire. The present results revealed absence of a significant difference in BOP and PD among the study groups, indicating that type of retainer wire had no significant effect on gingival health. A non-randomized cohort conducted in 2019 reported similar results [30]. Thus, it may be hypothesized that the most important factor affecting the gingival health of patients with orthodontic retainers is the patient compliance to oral hygiene protocols, rather than the type of retainer. However, the accuracy of this statement needs to be further confirmed in future studies.

Selection of the type of retainer wire by the orthodontist is a subjective matter. Aldrees et al. [31] in their in vitro study concluded that different combinations of wires and composite resins yielded different failure rates; however, all tested combinations had clinically acceptable strength. Although their results were different from our findings, adequate clinical strength of all wire-composite combinations in their study was in line with the present results. Arash et al. [32] demonstrated that ribbon-shaped retainer wires had lower rate of detachment than braided stainless steel retainer wires; although the clinical results of both retainers were the same. As explained earlier, it appears that the differences in strength of different retainers are not clinically significant. Egli et al. [33] in their clinical trial found no significant difference in failure rate of retainers bonded by the direct and indirect techniques. Considering the absence of a significant difference in failure rate of different retainer wires, and comparing the present results with those of Egli et al. [33] it may be hypothesized that the survival of orthodontic retainers is probably more related to the clinician’s performance in precise bonding of retainers rather than the bonding technique or wire type.

Retainer failures more commonly occur in the first 6 months after bonding, and patient’s age and operator’s experience reportedly have no significant effect on the frequency of failures [15]. Baysal et al. [34] reported higher failure rate of Bond-A-Braid wires compared with 0.0215-inch five-stranded wires, and 0.0195-inch dead-soft coaxial wires [34]. Also, Samson et al. [35] demonstrated the superior bond strength of 0.036-inch three-stranded twisted lingual retainer wires (3 M Unitek) compared with Bond-A-Braid. Nonetheless, the present study found no significant difference in failure rate of the three types of retainer wires. This difference may be due to the fact that both of the abovementioned studies had an in vitro design. Kocher et al. [21] evaluated braided stainless steel and TMA wires, and reported that wire detachment followed by composite damage were the most frequent first failures. Also, Salehi et al. [36] compared ribbon and spiral multi-stranded wires and reported that loosening of the retainer in both the maxilla and mandible was the most common type of failure; while, wire fracture in the maxilla and retainer loosening in the mandible were the most common types of failure in ribbon retainers. However, in the present study, wire fracture was the most common failure type in both jaws. This controversy in the results can be due to the differences in types of wires and composites. Also, the technique of retainer placement adopted by the clinician might have affected the results.

Another interesting finding of the present study was absence of unwanted tooth movements, and no caries development in teeth bonded to retainers, irrespective of the retainer type. Evidence shows higher prevalence of unwanted tooth movements in patients with oral and dental dysfunction. Also, unwanted tooth movements are more frequent in use of maxillary retainers [37]. Considering the significance of prevention of unwanted tooth movements, regular follow-ups are imperative after retainer placement [38]. No case of unwanted tooth movement in the present study can be due to regular follow-ups. Similarly, a previous study found no significant correlation between the presence of retainer or type of retainer wire with dental caries [17]. Moreover, another study indicated that presence of orthodontic retainers did not increase the occurrence of caries or periodontal disease [39]. Årtun et al. [40] in their clinical trial found no significant difference in PD, calculus index, or BOP of patients with different orthodontic retainers [40]. This finding is of particular interest since development of caries and periodontal disease is a common concern for most clinicians and patients using orthodontic retainers. Absence of dental caries, and good gingival and periodontal health can be due to regular follow-up visits and the great emphasis placed on oral hygiene maintenance during the follow-up period.

Only three types of wires were evaluated in the present study, which was a limitation of this study. Long-term clinical studies with larger sample size on higher number of retainer wires are required.

## Conclusion

The three types of retainer wires evaluated in this study were not significantly different in terms of survival rate in a 5-year period. Also, failure rate of retainers had no significant correlation with age or gender of patients or duration of treatment.

## Supplementary Information

**Additional file 1: **Mandibular retainers Data file.

**Additional file 2: **Maxillary retainers Data file.

**Additional file 3: **Mixed (Both arch) retainers Data file.

## Data Availability

All data generated or analysed during this study are included in this published article and its supplementary information files.

## Abbreviations

PD: Pocket depth
BOP: Bleeding on probing
